# Supplementary material for: Functional Prediction of trans-Prenyltransferases Reveals the Distribution of GFPPSs in Species beyond the Brassicaceae Clade
Source: Int J Mol Sci. 2022 Aug 22;23(16):9471. doi: 10.3390/ijms23169471 (PMC9409350; doi:10.3390/ijms23169471)
Supplement: Supplementary file 1 [file ijms-23-09471-s001.zip › ijms-1853303-supplementary.pdf]

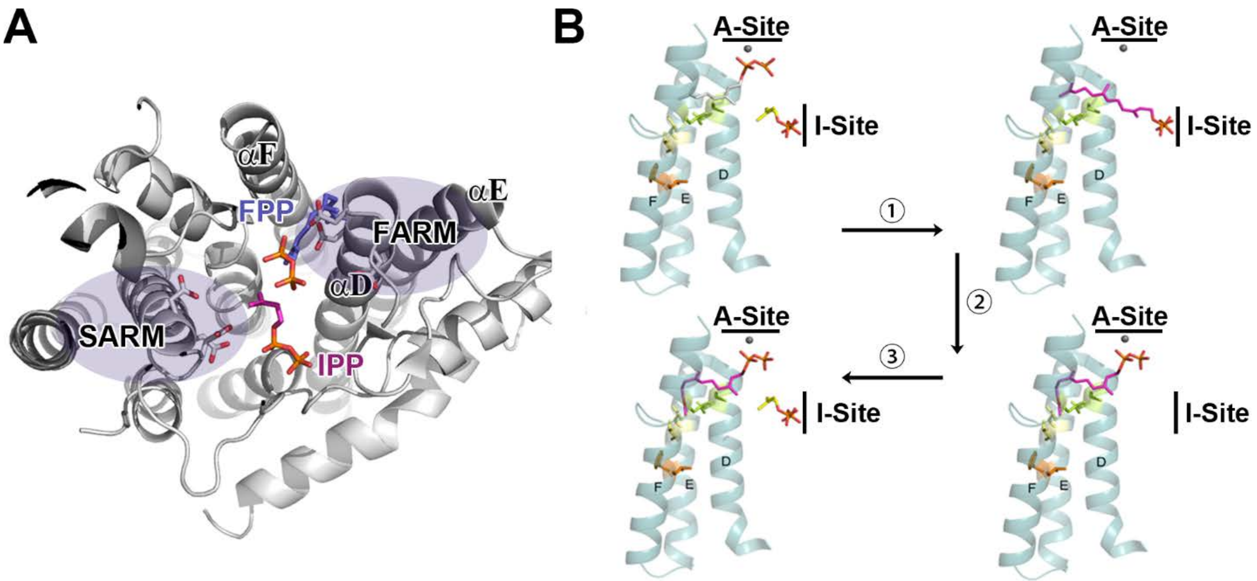

Figure S1 The overall structure and reaction mechanism of *trans*-PTs. (A) Overall structure of an *At*GFPPS2 monomer (top view). The structural cartoon is colored in grey. The FARM and SARM motifs, the IPP in I-site, the FPP in A site, and the product elongation pocket are indicated. **(B)** The mechanism of condensation reactions. The substrates IPP, GPP and FPP are shown with sticks and colored in yellow, grey and magenta, respectively.

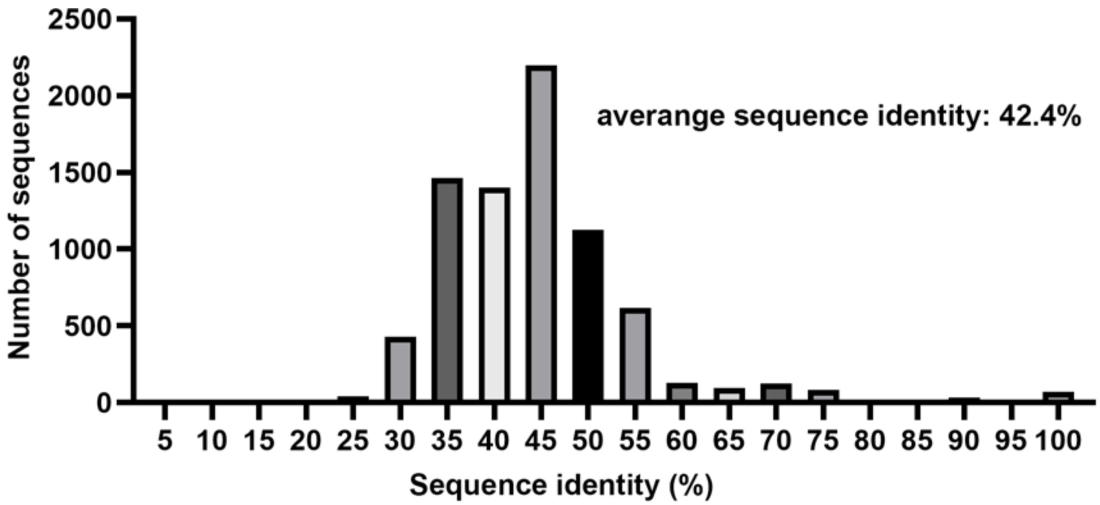

Figure S2 The distribution of sequence identity in the *trans*-PTs dataset. Each of the 7,870 *trans*-PTs was aligned with all the searching templates and the highest sequence identity was

taken into the calculation of the distribution. The average sequence identity of the whole dataset is also shown in the chat.

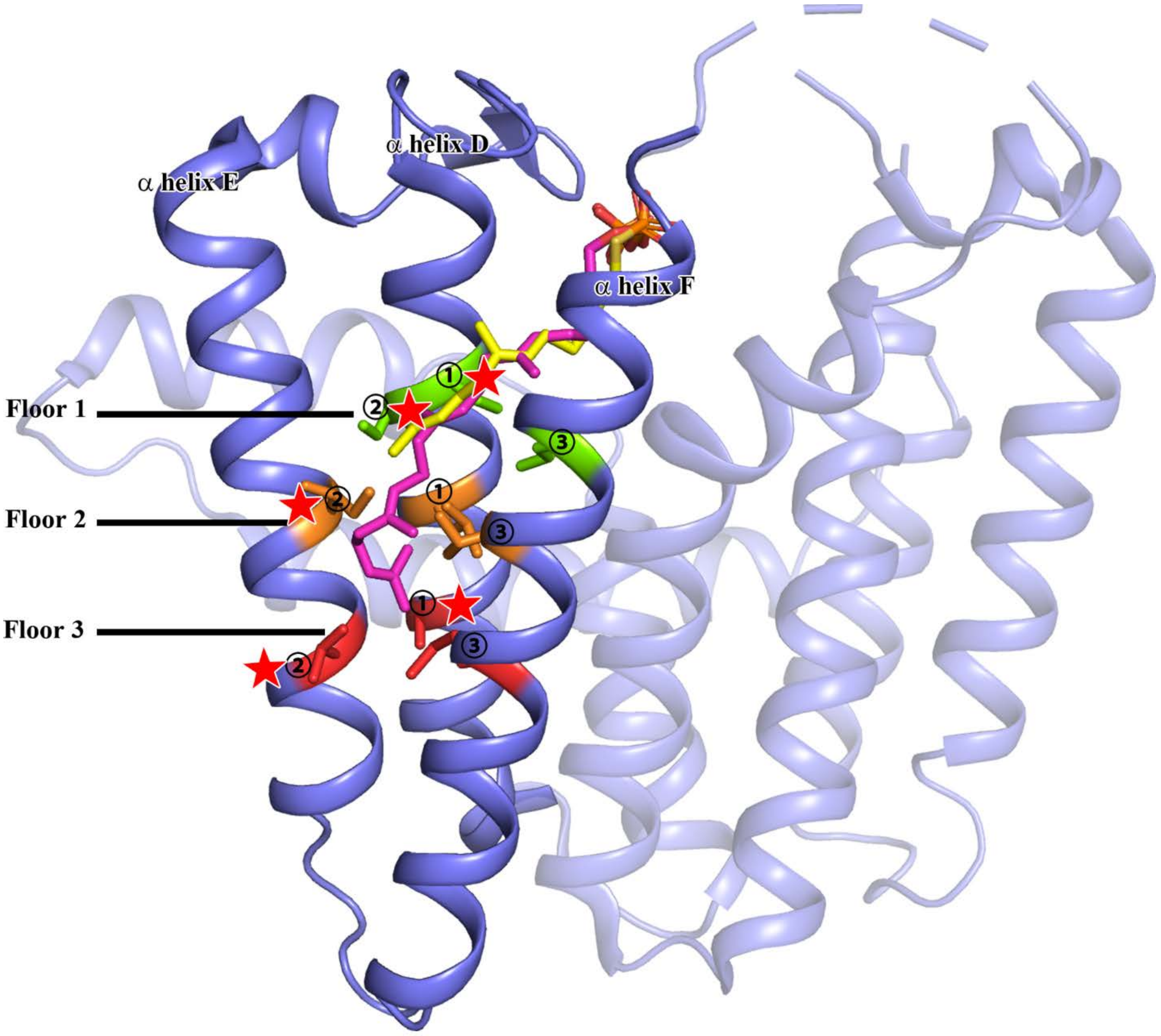

Figure S3. The proposed products' chain elongation route in *trans*-PTs. The "products' chain elongation route" is illustrated using *At*GGPPS11. A proposed GGPP model is shown with sticks and colored in magenta. The experimentally determined substrate FPP (yellow sticks) is also modeled into the structure using 3WJN. The three "floor" residues are highlighted with sticks and colored in green, orange and red, respectively. The "floor" residues with higher weight in the final "blocking" score are marked with red stars.

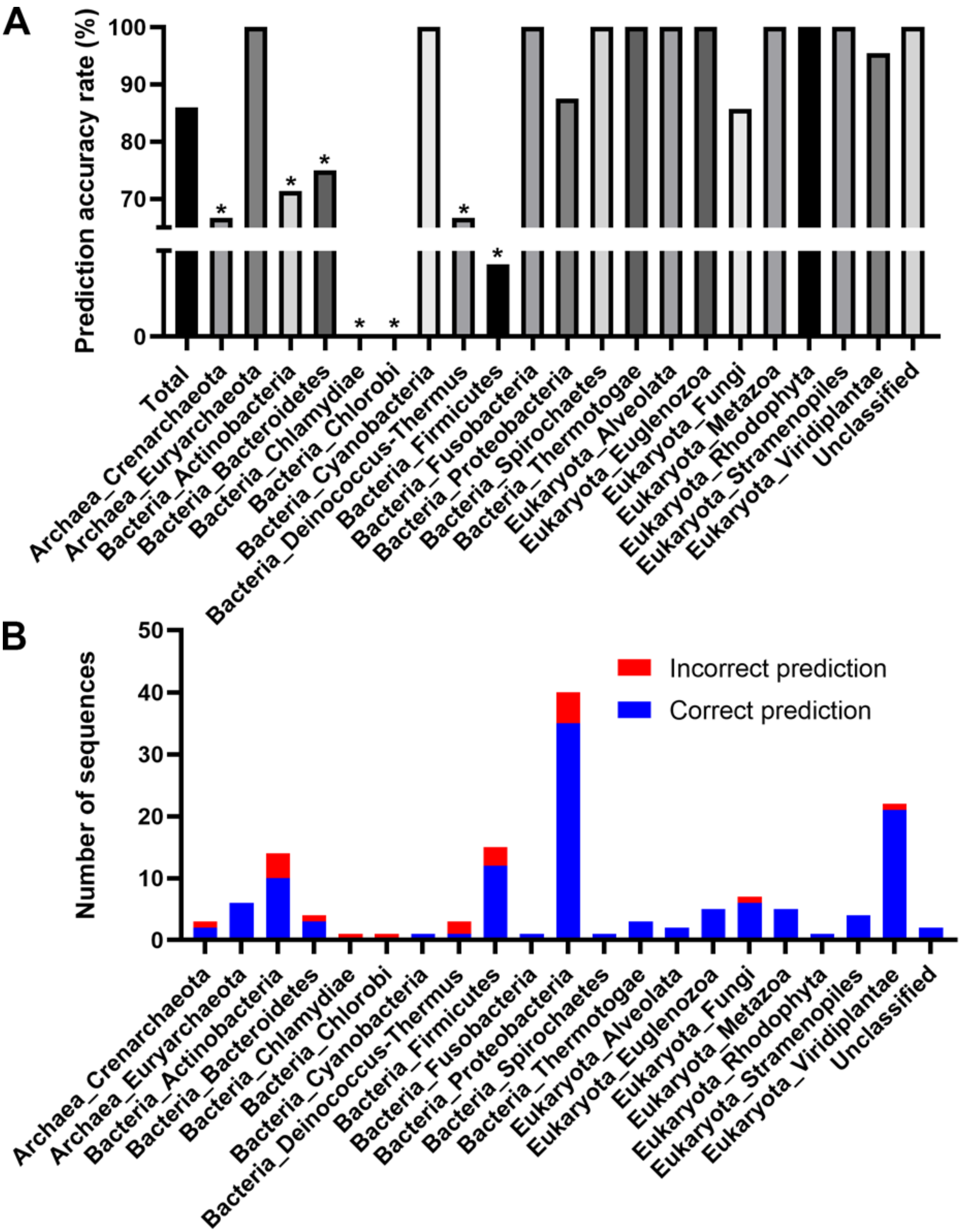

Figure S4. Prediction results for the 141 experimentally determined *trans*-PTs. (A)

Comparison of prediction accuracy by different species. Panels with comparatively higher or

lower accuracy are marked with asterisks. **(B)** The distribution of incorrect and correct prediction results in different species.

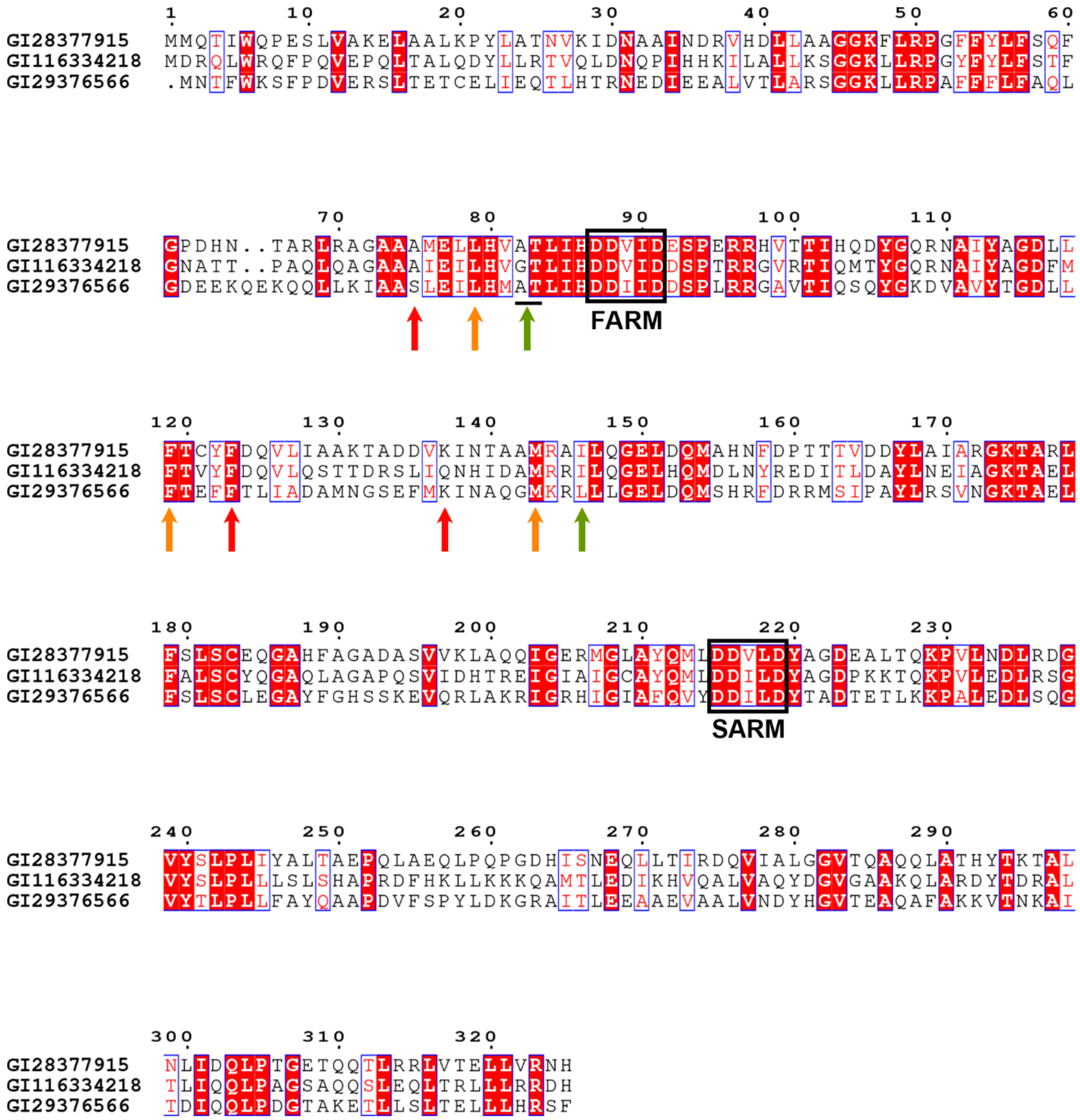

Figure S5. Sequence alignment of three proteins using 3PKO as template. The residues constituting the product elongation pocket helices and three floors are aligned. Invariant residues are highlighted in red, and conserved amino acids are boxed. The FARM and SARM motifs are

marked with black rectangular. The floor 1, 2 and 3 residues are indicated by green, orange, and red arrows, respectively.

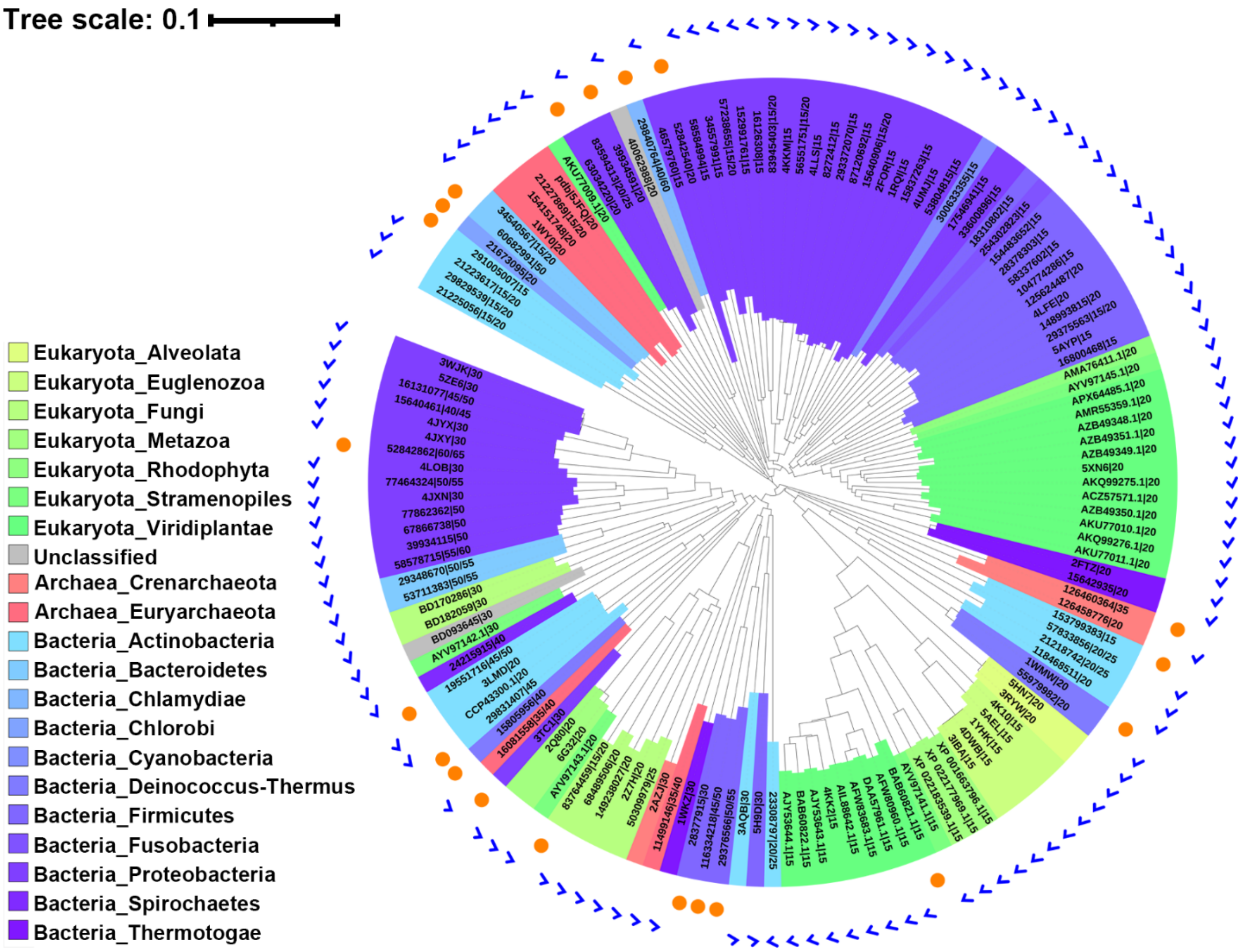

Figure S6 Phylogenetic analysis of the prediction results in different species. 141 experimentally determined *trans*-PTs protein sequences were used in constructing the neighbor-joining phylogenetic tree. The incorrectly and correctly predicted cases are marked as orange circles and blue checkmark, respectively.

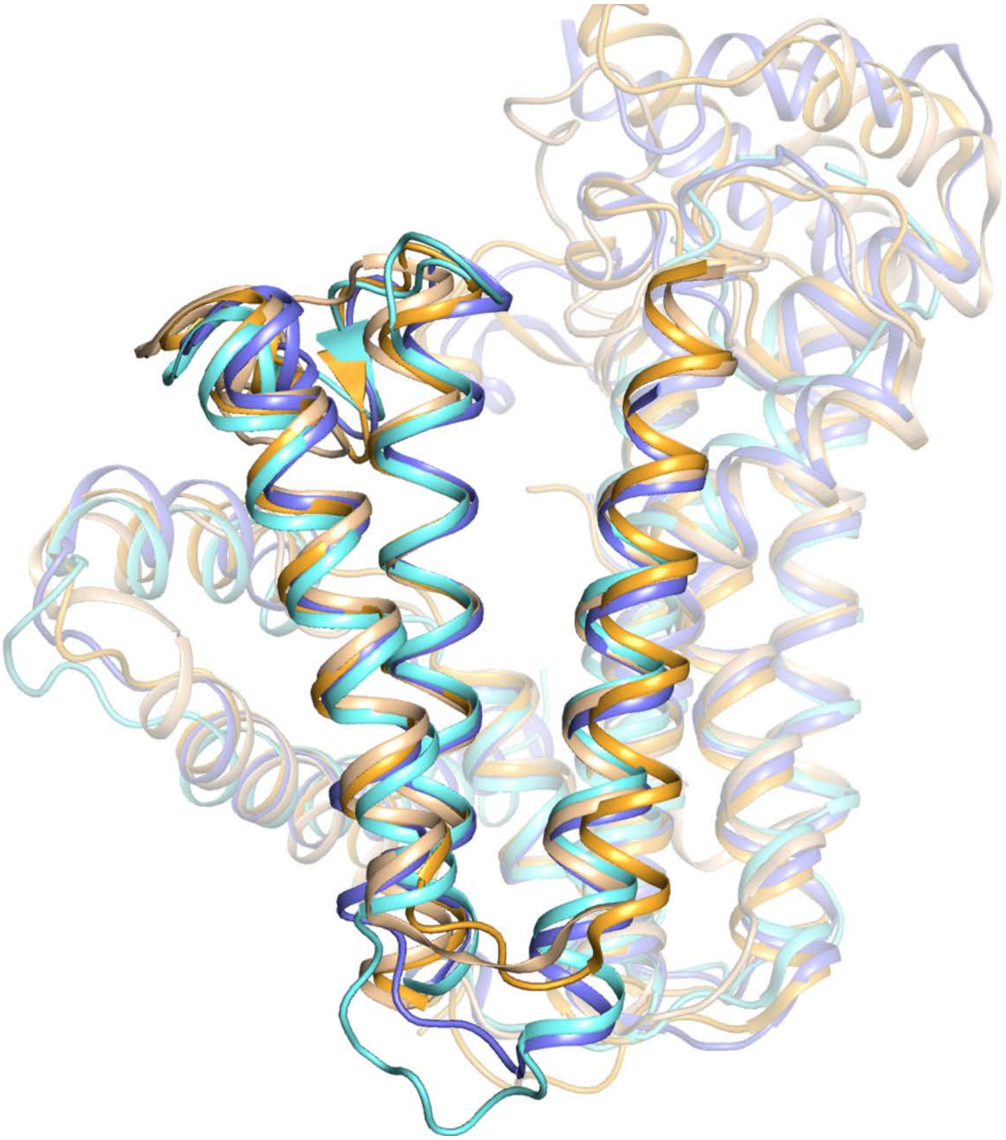

Figure S7 Superimposition of *trans*-PTs structures. Four *trans*-PTs structures (PDB IDs: 5H9D, 1WKZ, 1WY0, and 5E8L) are superimposed. The structures are shown with ribbons, and are colored in cyan (5E8L), orange (5H9D), wheat (1WKZ), and light blue (1WY0), respectively. The elongation tunnel is highlighted with other parts of the structures shown in transparent.

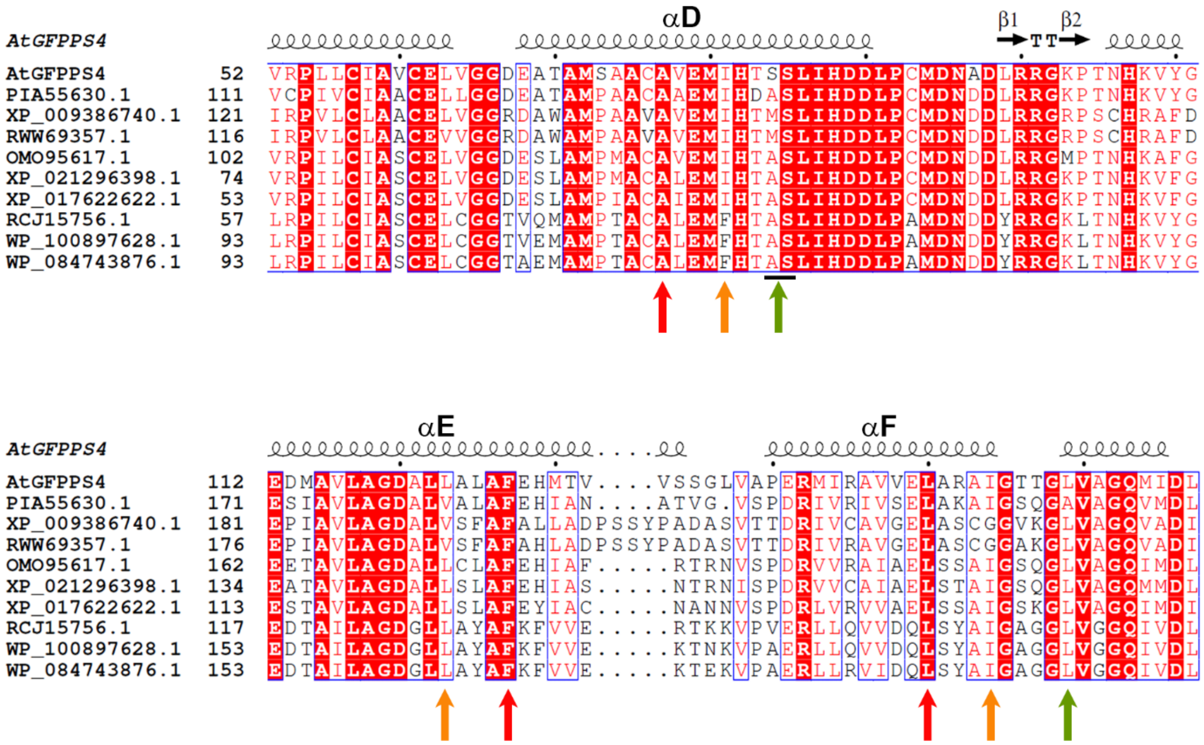

Figure S8. Comparison of "floor" residues in predicted and identified GFPPSs. The residues constituting the product elongation pocket helices and three floors are aligned. Invariant residues are highlighted in red, and conserved amino acids are boxed. The secondary structural elements of *At*GFPPS4 are shown above the aligned sequences. The floor 1, 2 and 3 residues are indicated by green, orange, and red arrows, respectively.

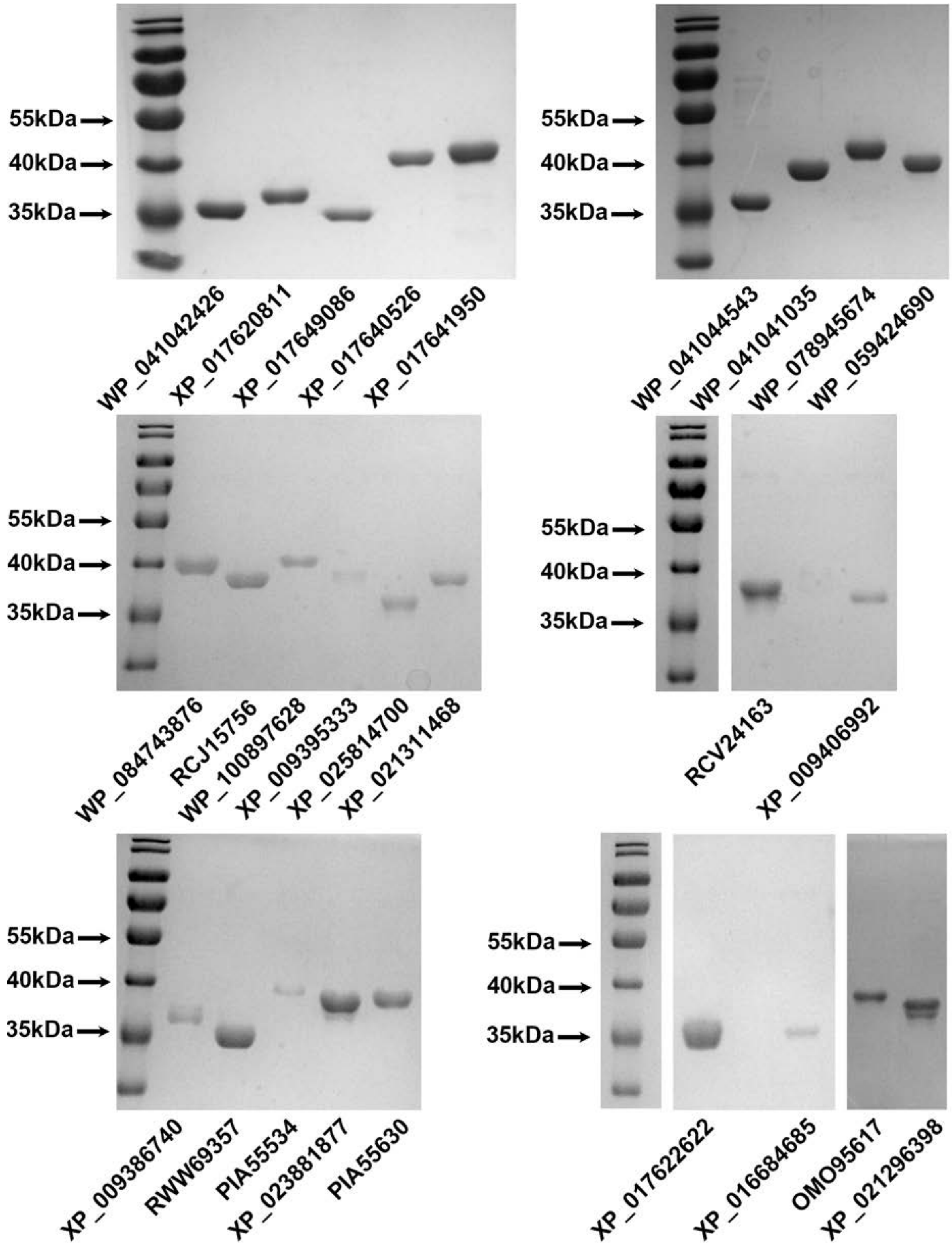
55kDa
40kDa
35kDa
WP_041042426
XP_017620811
XP_017649086
XP_017640526
XP_017641950
55kDa
40kDa
35kDa
WP_041044543
WP_041041035
WP_078945674
WP_059424690
55kDa
40kDa
35kDa
WP_084743876
RCJ15756
WP_100897628
XP_009395333
XP_025814700
XP_021311468
55kDa
40kDa
35kDa
RCV24163
XP_009406992
55kDa
40kDa
35kDa
XP_009386740
RWW69357
PIA55534
XP_023881877
PIA55630
55kDa
40kDa
35kDa
XP_017622622
XP_016684685
OMO95617
XP_021296398

Figure S9. The expression and purification of predicted GFPPSs. SDS-page gels show the purified proteins in different panels. The molecular weights are indicated by the protein markers. The 55 kDa, 40 kDa, 35 kDa bands of the marker are pointed by black arrows and labels.

Table S1. The "blocking score" for each amino acid.

Amino acid	"Blocking score"	Amino acid	"Blocking score"	Amino acid	"Blocking score"
Ala	105.27	Arg	146.71	Asn	120.11
Asp	120.58	Cys	108.69	Glu	132.12
Gln	135.4	Gly	100.33	His	140.57
Ile	121.04	Leu	121.33	Lys	129.88
Met	126.38	Phe	142.42	Pro	109.14
Ser	108.64	Thr	108.01	Trp	171.16
Tyr	148.79	Val	111.88		

Table S2. The weighting factors for each site of "floor" residues.

	Site_1 (Percentage for final blocking score of "floor")	Site_2 (Percentage for final blocking score of "floor")	Site_3 (Percentage for final blocking score of "floor")
"First floor"	0.7061 (48.7%)	0.7274 (50.2%)	0.0152 (1.1%)
"Second floor"	0.1288 (20.0%)	0.3566 (55.3%)	0.1592 (24.7%)
"Third floor"	0.5476 (36.5%)	0.9449 (62.9%)	0.0089 (0.6%)

Table S3. Experimentally determined product chain length of *trans*-PTs sequences from MD-Pre testing dataset.

GI	Experiment	Detection methods	MD-Pre predicted	PTS-Pre predicted
77862362	C50/C55	TLC	C25	≥C30
293372070	C15	TLC	C15	C15
19551716	C45/C50	TLC	C25	C20/C25
21225056	C15/C20	TLC	C20	C15
15640906	C15/C20	TLC	C15	C15
29375563	C15/C20	TLC	C15	C15
52842540	C20	TLC	C20	C15
57238655	C15/C20	TLC	C20	C15
116334218	C45/C50	TLC	≥C30	C20

8272412	C15	TLC	C15	C15
77464324	C50/C55	TLC	C25	≥C30
67866738	C50	TLC	C25	≥C30
39934115	C50	TLC	C25	≥C30
125624487	C20	TLC	C20	C20
16126308	C15	TLC	C20	C15
118468511	C20	TLC	C20	C20
148993815	C20	TLC	C20	C20
16131077	C45/C50	TLC	C25	≥C30
15640461	C40/C45	TLC	C20	≥C30
104774286	C15	TLC	C15	C15
21223617	C15/C20	TLC	C25	C15
29376566	C50/C55	TLC	C25	C20
52842862	C60/C65	TLC	C25	C20
21227869	C15/C20	TLC	C25	C20
21218742	C20/C25	TLC	C20	C20
58337602	C15	TLC	C15	C15
34540567	C15/C20	TLC	C15	C15
57833856	C20/C25	TLC	C25	C20
60682991	C50	TLC	C15	C20
29348670	C50/C55	TLC	C25	≥C30
153799383	C15	TLC	≥C30	≥C30
39934591	C20	TLC	C20	C25
53711383	C50/C55	TLC	≥C30	≥C30
23308797	C20/C25	TLC	C15	C15/C20
53804815	C15	TLC	C15	C15
18310802	C15	TLC	C15	C15
55979982	C20	TLC	C15	C20
15642935	C20	TLC	C20	C20
34557991	C15	TLC	C15	C15
28378303	C15	TLC	C15	C15
15837263	C15	TLC	C15	C15
87120692	C15	TLC	C15	C15
28377915	C30	TLC	C20	C20
33600896	C15	TLC	C20	C15
291005007	C15	TLC	C20	C20
152991761	C15	TLC	C15	C15
300633355	C15	TLC	C20	C15
17546941	C15	TLC	C15	C15
29831407	C45	TLC	≥C30	C20
46579760	C15	TLC	C15	C15
56551751	C15/C20	TLC	C15	C15
16800468	C15	TLC	C15	C15
68489506	C20	TLC	C20	C20
40062988	C20	TLC	C25	C20
50309979	C25	TLC	C25	C25

149238027	C20	TLC	C20	C20
154483652	C15	TLC	C15	C15
254302823	C15	TLC	C15	C15
83945403	C15/C20	TLC	C20	C15
63034220	C20	TLC	C20	≥C30
11499146	C35/C40	TLC	C25	≥C30
15805956	C40	TLC	C25	C20
58578715	C55/C60	TLC	C25	≥C30
83764459	C15/C20	TLC	C25	≥C30
83594313	C20/C25	TLC	C20	C25
154151748	C20	TLC	C25	C20/C25
58584994	C15	TLC	C15	C15
29829539	C15/C20	TLC	C20	C15
21673095	C20	TLC	C20	C15
16081558	C35/C40	TLC	C20	≥C30
29840764	C40-C60	TLC	C15	C25
126458776	C20	TLC	C20	C20
24215915	C40	TLC	C25	≥C30
126460364	C35	TLC	C25	C20/C25

The incorrect predictions are highlighted with light-orange and the correct predictions are highlighted with light blue.

Table S4. The number of predicted sequences by MD-Pre and PTS-Pre.

	PTS-Pre correctly predicted	PTS-Pre incorrectly predicted	Total
MD-Pre correctly predicted	37	6	43
MD-Pre incorrectly predicted	20	11	31
Total	57	17	

The incorrect predictions are highlighted with light-orange and the correct predictions are highlighted with light blue.

Table S5. Experimentally determined product chain length of *trans*-PTs sequences by using different detecting methods.

Protein or PDB ID	Experiment	Detection methods	PTS-Pre predicted	Ref (Pubmed ID)
APX64485.1	C20	HPLC-MS/MS	C20	30137453
CCP43300.1	C20	GC-MS	C20	30301210
BAB60822.1	C15	UPLC-MS/MS	C15	28478108
BAB60821.1	C15	UPLC-MS/MS	C20	28478108
ACZ57571.1	C20	GC-MS	C20	24346420
AZB49351.1	C20	GC-MS	C20	30448883
AZB49350.1	C20	GC-MS	C20	30448883
AZB49349.1	C20	GC-MS	C20	30448883
AZB49348.1	C20	GC-MS	C20	30448883
AKU77011.1	C20	GC-MS	C20	26449416
AKU77010.1	C20	GC-MS	C20	26449416
AKU77009.1	C20	GC-MS	C20	26449416
AKQ99276.1	C20	GC–MS	C20	26449416
AKQ99275.1	C20	GC–MS	C20	26449416
AMR55359.1	C20	β-carotene	C20	[37]
BD170286	≥C30	HPLC	≥C30	27837315
BD093645	≥C30	HPLC	≥C30	27837315
BD182059	≥C30	HPLC	≥C30	27837315
AFW83683.1	C15	GC–MS	C15	25680349
DAA57961.1	C15	GC–MS	C15	25680349
AFW80960.1	C15	GC–MS	C15	25680349
AYV97141.1	C15	GC–MS	C15	30394540
AYV97142.1	C25/C30	GC–MS	≥C30	30394540
AYV97143.1	C20	GC–MS	C20	30394540
AYV97145.1	C20	GC–MS	C20	30394540
XP_022183539.1	C15	GC–MS	C15	30569472
XP_022177969.1	C15	GC–MS	C15	30569472
AJY53644.1	C15	GC–MS	C15	25938487
AJY53643.1	C15	GC–MS	C15	25938487
XP_001663796.1	C15	HPLC	C15	26188328
AIL88642.1	C15	HPLC-MS	C15	[38]
AMA76411.1	C20	β-carotene	C20	[39]
1RQI	C15	TLC	C15	32584028
1WKZ	≥C30	TLC	≥C30	15984931
1WMW	C20	TLC	C15	10192906
1YHK	C15	TLC	C15	18096393
2FOR	C15	TLC	C15	32584028
2FTZ	C20	GC-MS	C20	25491272
1WY0	C20	GC-MS	C20	30062607
2AZJ	C30	TLC	C30	16291686
2Q80	C20	TLC	C20	16698791
2Z7H	C20	HPLC	C20	35077178
3IBA	C15	TLC	C15	18096393
3LMD	C20	HPLC	C20/C25	25181035

3AQB	≥C30	TLC	≥C30	21068379
3TC1	≥C30	TLC	C20	24895191
3RYW	C20	HPLC	C15/C20	30275041
4DWB	C15	TLC	C15	18096393
4JYX	≥C30	TLC	≥C30	8765142
4JXY	≥C30	TLC	≥C30	8765142
4JXN	≥C30	TLC	≥C30	29191106
4KKM	C15	TLC	C15	33732763
4KK2	C15	TLC	C15	33732763
4LFE	C20	TLC	C20	19690851
4LOB	≥C30	HPLC	≥C30	31354766
4LLS	C15	HPLC	C15	2676985
4K10	C15	TLC	C15	17724033
3WJK	≥C30	TLC	≥C30	24895191
4UMJ	C15	ESI-MS	C15	25760619
5AEL	C15	HPLC-MS	C15	26392508
5HN7	C20	HPLC	C15/C20	27564465
5AYP	C15	TLC	C15	8755734
5H9D	≥C30	TLC	≥C30	30730737
5XN6	C20	LC-MS/MS	C20	28607067
6G32	C20	HPLC	C20	30275041
5ZE6	≥C30	TLC	≥C30	30730737
5JFQ	C20	GC-MS	C20	30062607

References

37. Shang, C.H.; Xu, X.L.; Yuan, Z.H.; Wang, Z.M.; Hu, L.; Alam, M.A.; Xie, J. Cloning and differential expression analysis of geranylgeranyl diphosphate synthase gene from Dunaliella parva. J. Appl. Phycol. 2016, 28, 2397–2405.

38. Qi, Q.; Li, R.; Gai, Y.; Jiang, X.N. Cloning and functional identification of farnesyl diphosphate synthase from Pinus massoniana Lamb. J. Plant Biochem. Biot. 2017, 26, 132–140.

39. Yang, L.E.; Huang, X.Q.; Lu, Q.Q.; Zhu, J.Y.; Lu, S. Cloning and characterization of the geranylgeranyl diphosphate synthase (GGPS) responsible for carotenoid biosynthesis in Pyropia umbilicalis. J. Appl. Phycol. 2016, 28, 671–678.
